# Blood pressure trajectories during pregnancy, labor, and postpartum period: a multicenter study in Japan

**DOI:** 10.1038/s41440-026-02708-3

**Published:** 2026-06-10

**Authors:** Takafumi Ushida, Takuma Shimaya, Tatsuo Inamura, Satoru Katsuki, Sho Tano, Seiko Matsuo, Kazuya Fuma, Shigeru Yoshida, Mamoru Yamashita, Hiroaki Kajiyama

**Affiliations:** 1 Department of Obstetrics and Gynecology, Nagoya University Graduate School of Medicine, Nagoya, Japan; 2 Division of Reproduction and Perinatology, Center for Maternal-Neonatal Care, Nagoya University Hospital, Nagoya, Japan; 3 Kishokai Medical Corporation, Nagoya, Japan

**Keywords:** Blood pressure, Labor, Postpartum, Pregnancy

## Abstract

In this study, we characterized the physiological blood pressure (BP) trajectories from an estimated pre-pregnancy period through pregnancy, labor, and postpartum among low-risk, normotensive women. This retrospective multicenter observational study was conducted at 12 primary obstetric care facilities in Japan and included 14,240 low-risk, normotensive women with term singleton deliveries between 2011 and 2018. Longitudinal outpatient BP trajectories during pregnancy and the postpartum period were modeled using linear mixed-effects models with restricted cubic splines. The pre-pregnancy BP was approximated using 1:1 matching with non-pregnant women undergoing routine health checkups. Changes in intrapartum BP were evaluated in women who underwent vaginal delivery (*n* = 11,836). The outpatient BP followed a classic gestational pattern, with a nadir at approximately 25 weeks of gestation, followed by a gradual increase toward term. The estimated pre-pregnancy BP was lower than the outpatient BP measured during early pregnancy. During the first stage of labor, systolic and diastolic BPs increased by up to 15.7 mmHg and 13.4 mmHg, respectively, compared with the final outpatient measurements, returning to near-outpatient levels within 2 h after delivery. During the postpartum period, the BP increased, peaked at days 5–7, and then gradually declined by approximately 5 weeks, converging toward non-pregnant levels. Low-risk normotensive women demonstrated elevated BP in early pregnancy relative to the estimated non-pregnant baseline during labor and in the first postpartum week. These data provide physiological reference patterns that may aid the interpretation of intrapartum and postpartum hypertension.

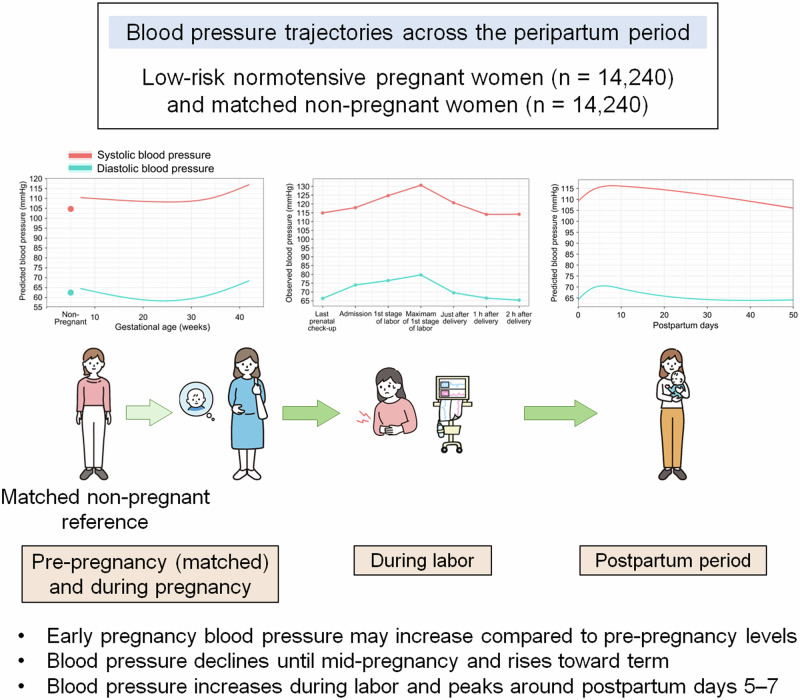

## Introduction

Pregnancy elicits profound cardiovascular adaptations to accommodate the increasing metabolic demands of both the mother and the developing fetus [1–3]. Circulating plasma volume and cardiac output progressively increase as gestation advances, reaching approximately 1.5-fold higher levels than those observed in the non-pregnant state by the third trimester [1]. These hemodynamic changes are accompanied by a reduction in systemic vascular resistance mediated by hormonal and endothelial mechanisms, most notably, nitric oxide–dependent vasodilation. Consequently, blood pressure (BP) typically declines from early to mid-pregnancy, reaches a nadir at approximately 20 weeks of gestation, and gradually increases toward delivery [1, 4]. A clear understanding of these physiological BP trajectories is essential for distinguishing normal cardiovascular adaptations from pathological conditions, including hypertensive disorders of pregnancy.

Most studies have focused predominantly on BP changes during pregnancy [5, 6]; the BP dynamics during labor and the postpartum period, which are characterized by rapid and pronounced cardiovascular shifts, remain unclear [7]. Labor constitutes an intense physiological stress state involving pain, sympathetic nervous system activation, catecholamine release, uterine contractions, maternal expulsive efforts, and anxiety [2, 8]. Each of these factors may provoke acute elevations in BP, even in women who remain normotensive throughout pregnancy. Following delivery, the intravascular volume transiently increases because of the mobilization of interstitial fluid into the vascular compartment [2]. Additionally, nonsteroidal anti-inflammatory drug use, breastfeeding-related neuroendocrine changes, sleep deprivation, and psychological stress may further influence BP regulation through heightened sympathetic activity [9, 10]. Although intrapartum and postpartum hypertension have been increasingly recognized as clinically important conditions [11, 12], longitudinal data describing the trajectory of intrapartum BP elevation and subsequent postpartum BP normalization in normotensive women remain limited.

Another challenge in evaluating BP trajectories during pregnancy is the frequent absence of reliable baseline pre-pregnancy measurements. In routine obstetric practice, most women present for care only after conception, rendering true pre-pregnancy BP data rarely available. Consequently, BP measured during early pregnancy is often used as a surrogate for pre-pregnancy values. Although modest reductions in BP from the non-pregnant state to early pregnancy have been reported [4, 13], substantial evidence has been derived from relatively small cohorts and studies conducted decades ago [4, 14]. Therefore, whether BP measured during early pregnancy reflects unaltered pre-pregnancy levels or incorporates pregnancy-related physiological adaptations remains uncertain.

To date, large-scale longitudinal datasets capturing BP trajectories from the pre-pregnancy period through pregnancy, labor, and the postpartum recovery phases are limited. Previous studies have been limited by small sample sizes, short observation periods, and the substantial inclusion of high-risk populations. Moreover, differences in healthcare systems, antenatal care practices, and population characteristics limit the generalizability of the findings from international cohorts to the Japanese population.

Therefore, we conducted a retrospective multicenter observational study to characterize the BP trajectories across the entire peripartum period in low-risk, normotensive women. Using clinical data from women who delivered at term in primary obstetric care settings, we modeled longitudinal changes in outpatient BP during pregnancy and the postpartum period using linear mixed-effects models that incorporated restricted cubic splines. To compensate for the absence of true pre-pregnancy BP measurements, we employed a pseudo-longitudinal approach using a non-pregnant reference population matched for age, height, weight, smoking status, and drinking status to estimate pre-pregnancy BP. Additionally, changes in intrapartum BP were evaluated in women who underwent vaginal delivery.

We aimed to characterize the BP trajectories from the estimated pre-pregnancy period through pregnancy, labor, and postpartum recovery, thereby improving our understanding of physiological BP adaptations across the peripartum period in low-risk, normotensive women.

## Methods

### Study design and data sources

This retrospective multicenter observational study used clinical data routinely collected during standard obstetric care at 12 primary obstetric care facilities in Japan. The primary objective of this study was to characterize physiological BP trajectories from the estimated pre-pregnancy period through pregnancy, labor, and the postpartum period among low-risk, normotensive women. All participating facilities belonged to the same medical group and shared largely standardized clinical systems, obstetric care policies, antenatal and intrapartum management protocols, medical devices, and BP measurement procedures. Clinical records were managed using a common electronic medical record system from which anonymized data collected as part of routine clinical practice were extracted for analysis. The study protocol was approved by the Institutional Review Board of Nagoya University School of Medicine (approval number: 2015–0415), as well as by the ethics committees of Kishokai Medical Corporation and the Central Clinic Group. The requirement for informed consent was waived because all data were retrospectively collected and anonymized prior to analysis.

### Study population

Eligible participants were women who delivered at term (37 weeks, 0 days to 41 weeks and 6 days of gestation) at one of the participating facilities between 2011 and 2018 and had BP measurements available at four predefined time points: the first trimester (6–12 weeks of gestation), second trimester (13–27 weeks), third trimester (28–41 weeks), and postpartum period (2–7 weeks after delivery). To ensure a consistent longitudinal assessment of BP trajectories within individuals and minimize bias due to missing measurements across gestational stages, only cases with data available at all four time points were included in the analysis.

The exclusion criteria were multiple gestations; stillbirth; major fetal anomalies; missing clinical data on maternal age, height, or pre-pregnancy weight; preexisting medical conditions potentially affecting BP regulation, including hypertension, cardiovascular disease, renal disease, thyroid disorders, hematologic disease, diabetes mellitus, dyslipidemia, and liver disease; obstetric complications such as gestational diabetes mellitus, hypertensive disorders of pregnancy, placental abruption, placenta previa, or low-lying placenta; and delivery of infants classified as small- or large-for-gestational-age [15]. After applying these criteria, the final analytical cohort comprised 14,240 low-risk, normotensive women who delivered singleton infants at term.

### Blood pressure measurements during pregnancy, labor, and postpartum

Among the final analytic cohort, 188,903 outpatient BP measurements were recorded during pregnancy, corresponding to 13.3 ± 1.6 measurements per woman (mean ± standard deviation). Intrapartum BP data were analyzed for 11,836 women who underwent vaginal delivery. BP measurements were obtained at hospital admission (*n* = 11,579), during the first stage of labor (*n* = 9856), at the maximum value recorded during the first stage of labor (*n* = 3920), immediately after delivery (*n* = 11,608), and during the early postpartum period (1 h [*n* = 11,530] and 2 h [*n* = 11,389] after delivery). The interval between the final antenatal visit and hospital admission was 3.3 ± 3.1 days (mean ± standard deviation).

Postpartum BP data included measurements obtained during hospitalization and after discharge. In-hospital postpartum BP measurements were available for 12,276 women, yielding 50,799 measurements (4.1 ± 0.6 measurements per woman). When multiple measurements were recorded on the same day, the values obtained during the day shift were prioritized, followed by the evening- and night-shift measurements, if necessary. After discharge, 26,819 BP measurements were obtained from 14,240 women (1.9 ± 0.3 measurements per woman).

For the sub-analyses, BP trajectories during pregnancy, labor, and the postpartum period were stratified by parity (nulliparous vs. multiparous), and postpartum BP trajectories were stratified by mode of delivery (cesarean section vs. vaginal delivery).

Outpatient BP measurements were obtained using an automated arm BP monitor (TM-2657P; A&D Medical, Tokyo, Japan), whereas in-hospital BP measurements were obtained using an automated sphygmomanometer with a double cuff (Terumo ES-H55; Terumo Corporation, Tokyo, Japan).

### Non-pregnant reference population

To estimate the pre-pregnancy BP, clinical data of non-pregnant women who underwent routine medical checkups between 2016 and 2019 at a medical clinic in Aichi Prefecture, Japan (*n* = 63,095) were obtained. This clinic conducts comprehensive medical examinations of approximately 30,000 individuals annually. When two BP measurements were obtained during a single visit, the mean value was used. Women with a history of hepatobiliary or pancreatic disease, hypertension, myocardial infarction, cardiovascular disease, stroke, diabetes mellitus, dyslipidemia, hematological disease, thyroid disease, renal disease, or connective tissue disease were excluded. Women with the potential for pregnancy were also excluded, resulting in 54,905 eligible non-pregnant women for matching.

### Matching procedure

To approximate the pre-pregnancy BP, pregnant women were matched with non-pregnant women undergoing routine health checkups using propensity score matching. The propensity score was estimated using a logistic regression model that included maternal age, height, pre-pregnancy weight, smoking status, and drinking status. Propensity score matching was performed using greedy nearest-neighbor 1:1 matching with a caliper width of 0.25, which is the standard deviation of the logit of the propensity score. To minimize the repeated use of controls while retaining all pregnant participants, the maximum number of reuses per control was sequentially increased from 1, and the smallest value that allowed complete matching of all pregnant participants was selected. Covariate balance after matching was assessed using standardized mean differences, with values < 0.1 considered indicative of adequate balance. Matching was performed using the MatchIt package in R (version 4.5.2). Using this procedure, 14,240 matched pairs were generated and used as the pseudo-pre-pregnancy reference population for subsequent analyses.

### Sample size

No formal a priori sample size calculation was performed because this was a retrospective study that used routinely collected clinical data. All eligible women who delivered during the study period and met the predefined inclusion and exclusion criteria were included, yielding an analytical cohort of 14,240 low-risk, normotensive women with repeated BP measurements during pregnancy, labor, and the postpartum period. Although BP measurements were not uniformly distributed across all gestational weeks and postpartum days, the sample size and number of repeated measurements were considered sufficient to estimate population-level BP trajectories in routine clinical practice. The estimates for periods with relatively sparse measurements were interpreted with caution.

### Statistical analysis

Longitudinal BP trajectories were analyzed using linear mixed-effects models to account for repeated measurements within individuals and the unbalanced structure of the data. To flexibly model the temporal changes in BP, restricted cubic splines with three degrees of freedom (df = 3) were applied to the time variable [16]. Sensitivity analyses using alternative spline specifications with four and five degrees of freedom produced similar trajectories, indicating robustness to spline complexity (data not shown). Gestational age (weeks) was used as the spline variable for pregnancy analysis, and days since delivery were used for postpartum analysis. Restricted cubic splines were used to allow smooth and continuous modeling of BP trajectories while reducing the risk of overfitting by enforcing linearity beyond the boundary knots. All of the models included random intercepts for each individual to account for within-subject correlations arising from repeated measurements. Fixed-effect covariates were pre-specified based on established associations with BP and included maternal age and pre-pregnancy body mass index. Model-estimated BP trajectories represent population-averaged (fixed-effect) predictions corresponding to the expected mean BP profile for a hypothetical individual with covariates fixed at their median values. Predictions were generated using only fixed effects (random effects were set to zero). The model-estimated mean BP values and the corresponding 95% confidence intervals (CIs) were derived from the fixed effects, representing the uncertainty in the estimated population-averaged trajectories rather than the prediction intervals for individual measurements. Temporal changes in BP were interpreted based on model-derived estimates rather than raw observations, allowing for robust inference despite inter-individual variability in measurement timing and frequency. In contrast, time-continuous longitudinal data were not available for intrapartum BP, precluding the estimation of model-based trajectories using the same approach. Instead, the observed mean BP values and the corresponding 95% CIs were plotted. Linear mixed-effects models were fitted using the lme4 package in R software (version 4.5.2).

### Language proofreading

ChatGPT (OpenAI, GPT-5.3) was used exclusively for English-language proofreading.

## Results

**Fig. 1:**
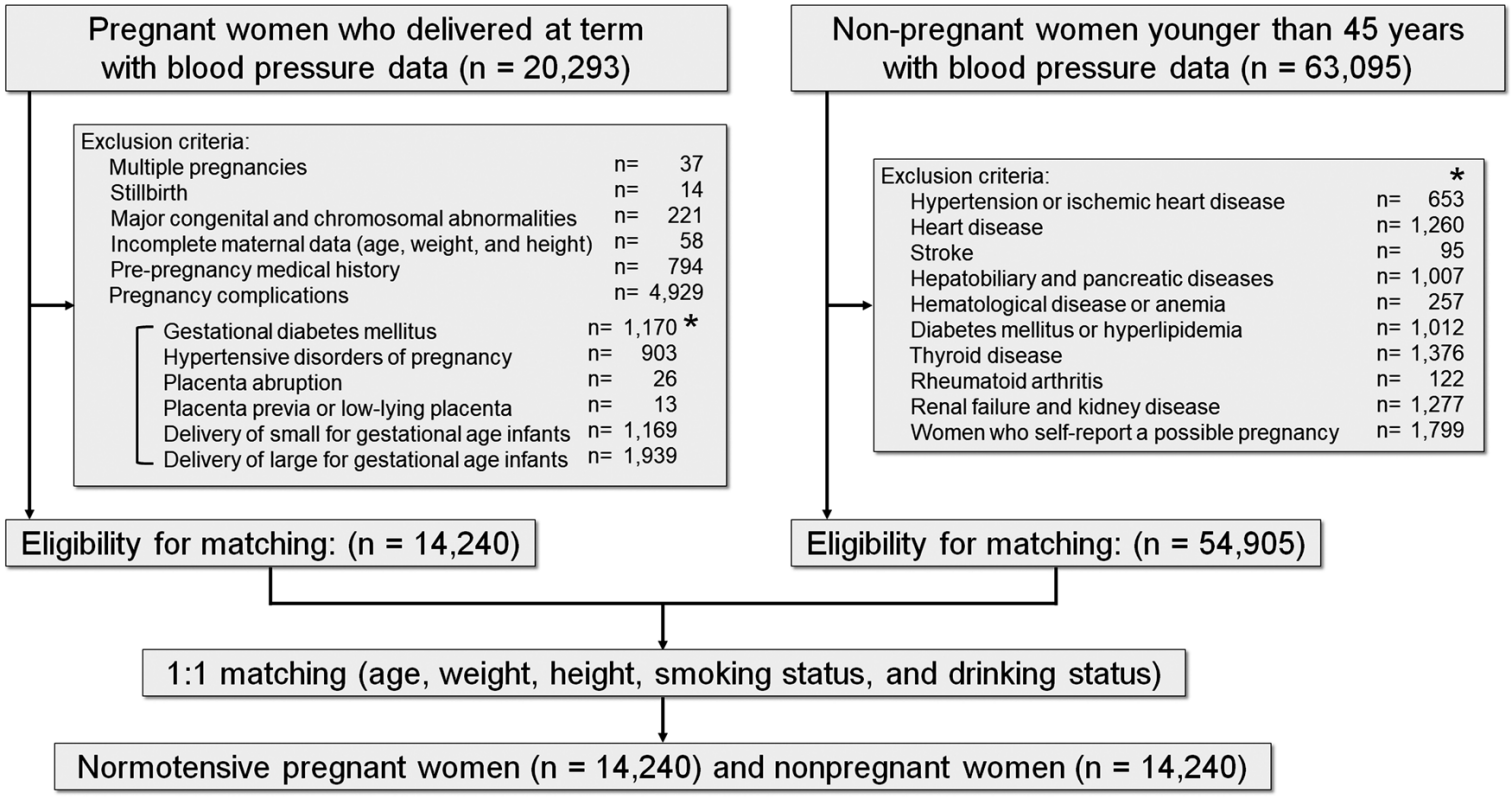
Flow diagram of the study population selection and matching procedure. Flow diagram illustrating the selection of the study population and matching procedures. Of the 20,293 pregnant women who delivered at term and had valid blood pressure measurements, pregnancies were sequentially excluded based on the gestational age criteria, multiple gestations, stillbirth or major fetal anomalies, missing maternal background data, preexisting medical conditions affecting blood pressure regulation, and obstetric complications. The final analytical cohort comprised 14,240 low-risk, normotensive women with singleton term deliveries. Non-pregnant women younger than 45 years with blood pressure data from routine health checkups were screened as a reference population, and eligible low-risk, normotensive pregnant women were matched 1:1 with non-pregnant women (*n* = 54,905) based on age, height, pre-pregnancy weight, smoking status, and drinking status. *Items are not mutually exclusive

A total of 20,293 pregnancies with valid BP measurements at the four pre-specified time points were identified. After applying the exclusion criteria, the final analytic cohort for the primary analysis comprised 14,240 normotensive women who delivered singleton infants at term (Fig. 1). For analyses incorporating the estimated pre-pregnancy BP, all 14,240 women were successfully matched 1:1 with non-pregnant women (*n* = 54,905) based on age, height, pre-pregnancy weight, smoking status, and drinking status. After matching, the baseline characteristics were well balanced between the pregnant and matched non-pregnant reference groups across all matching variables, with standardized mean differences of < 0.1 for all covariates (Table 1). The distribution of BP measurements during pregnancy and the postpartum period is shown in Supplementary Fig. 1.

**Table 1:** Baseline characteristics of pregnant women and propensity score–matched non-pregnant women

	Pregnant women	Non-pregnant women	Standardized mean difference
Characteristics	*n* = 14,240	*n* = 14,240	
Maternal characteristics			
Maternal age (years)	31.1 ± 4.6	30.9 ± 6.8	0.045
Gestational age at delivery (weeks)	39.5 ± 1.1		
Cesarean section (%)	2404 (16.9)		
Primipara (%)	6431 (45.2)		
Pre-pregnancy body weight (kg)	51.5 ± 7.6	51.7 ± 8.3	−0.018
Height (cm)	157.8 ± 5.3	157.9 ± 5.2	−0.025
ART	666/11,464 (5.8)		
Smoking before pregnancy	1150/11,075 (10.4)	1035/11,106 (9.3)	0.030
Drinking before pregnancy	3697/11,023 (33.5)	3689/11,005 (33.5)	0.001
Neonatal characteristics			
Male (%)	7227 (50.8)		
Birth weight (g)	3050 ± 274		

Data are shown as the mean ± standard deviation or *n* (%). Non-pregnant women represented a propensity score–matched reference cohort selected using 1:1 nearest-neighbor matching based on maternal age, height, pre-pregnancy body weight, smoking status, and drinking status. Standardized mean differences were used to assess covariate balance after matching

*ART* Assisted reproductive technology

**Fig. 2:**
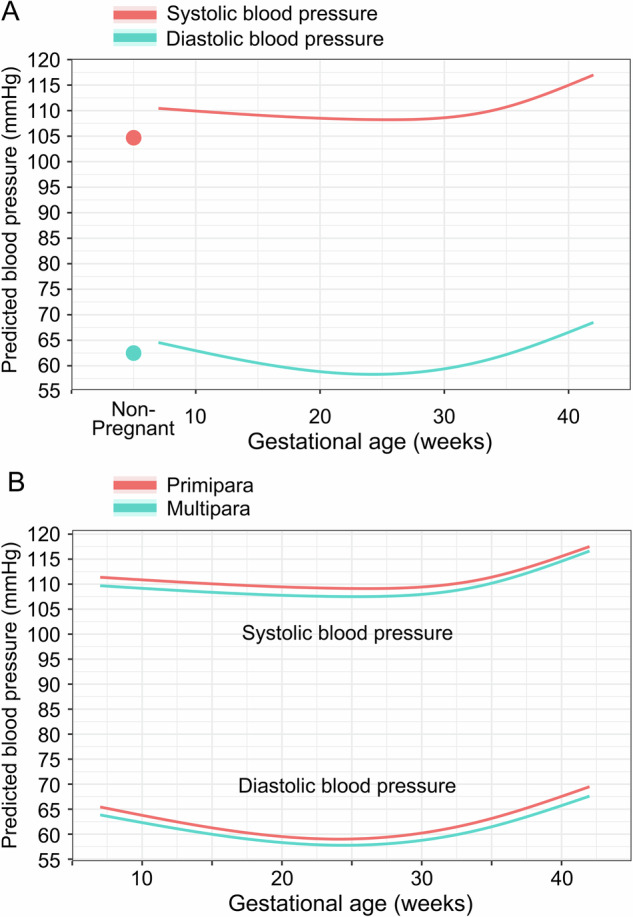
Model-estimated blood pressure trajectories during pregnancy and estimated pre-pregnancy blood pressure. Modeled outpatient blood pressure trajectories during pregnancy and estimated pre-pregnancy blood pressure in low-risk normotensive women. **A** Red and blue solid lines represent the model-estimated mean systolic and diastolic blood pressures, respectively, derived from linear mixed-effects models incorporating restricted cubic splines (df = 3). Shaded areas indicate 95% confidence intervals. Gestational age (in weeks) was used as the time variable. The estimated pre-pregnancy blood pressure values are shown as dots and were derived from non-pregnant women matched for age, height, pre-pregnancy weight, smoking status, and drinking status (red and blue dots represent systolic and diastolic blood pressure, respectively). **B** The model-estimated blood pressure trajectories of the nulliparous (red line) and multiparous (blue line) women are shown

Linear mixed-effects models incorporating restricted cubic splines revealed distinct nonlinear trajectories for both systolic and diastolic BPs during gestation (Fig. 2A; systolic BP is shown in red, and diastolic BP in blue). The systolic BP gradually declined from early pregnancy, reached a nadir at approximately 25 weeks of gestation, and subsequently increased gradually toward term delivery. Parity-stratified model-estimated BP trajectories demonstrated that nulliparous women (red line) exhibited slightly higher BP levels than multiparous women (blue line) (Fig. 2B).

As true pre-pregnancy BP measurements were unavailable, BP values from matched non-pregnant women were used as proxies for pre-pregnancy BP. The estimated pre-pregnancy BP was 105.4 mmHg (95% CI: 105.2–105.6 mmHg) for systolic BP and 62.5 mmHg (95% CI: 62.4–62.7 mmHg) for diastolic BP (Fig. 2). Unexpectedly, both systolic and diastolic BP values were lower than those observed during early pregnancy in the outpatient setting.

**Fig. 3:**
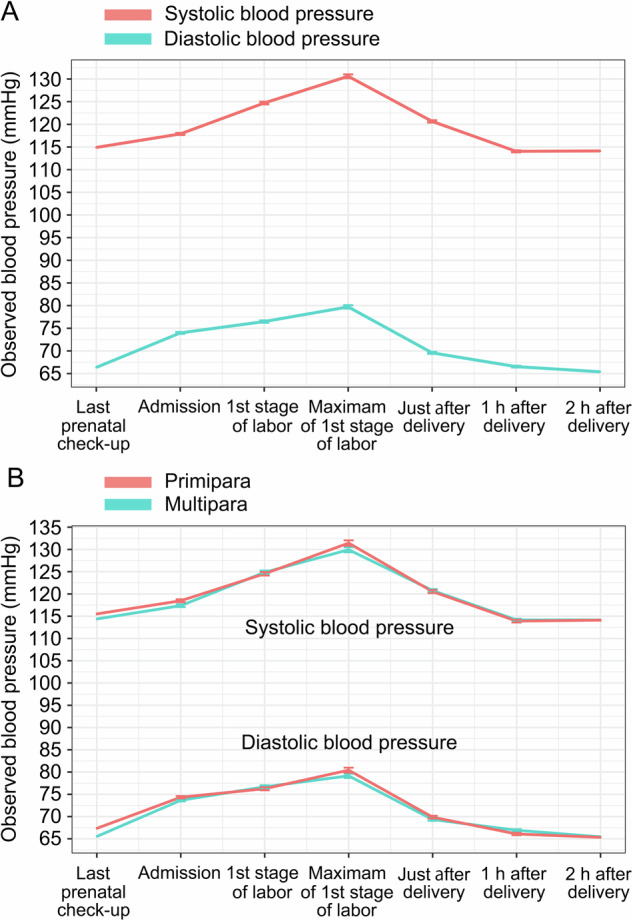
Blood pressure changes during labor. **A** Changes in blood pressure during labor in women who underwent vaginal delivery (*n* = 11,836). Red and blue lines indicate the mean systolic and diastolic blood pressures, respectively, with error bars representing 95% confidence intervals. Blood pressure was evaluated at the final outpatient visit, at hospital admission, during the first stage of labor, at peak intrapartum levels, immediately after delivery, and in the early postpartum period (1 and 2 h after delivery). **B** Changes in blood pressure during labor in nulliparous (red line) and multiparous (blue line) women

Among the 11,836 women who underwent vaginal delivery, BP increased substantially during labor (Fig. 3A; systolic BP is shown in red and diastolic BP in blue). Compared with the values at the final outpatient visit, the systolic and diastolic BPs increased by 3.0 mmHg and 7.6 mmHg, respectively, at hospital admission. During the first stage of labor, systolic and diastolic BPs increased by 9.8 mmHg and 10.1 mmHg, respectively, compared with those at the final outpatient visit. The peak increases in systolic and diastolic BP were 15.7 mmHg and 13.4 mmHg, respectively. The BP declined to levels comparable to those observed at the final outpatient visit within 2 h after delivery. The parity-stratified BP trajectories during labor are presented in Fig. 3B (nulliparous women are indicated with red lines and multiparous women with blue lines).

**Fig. 4:**
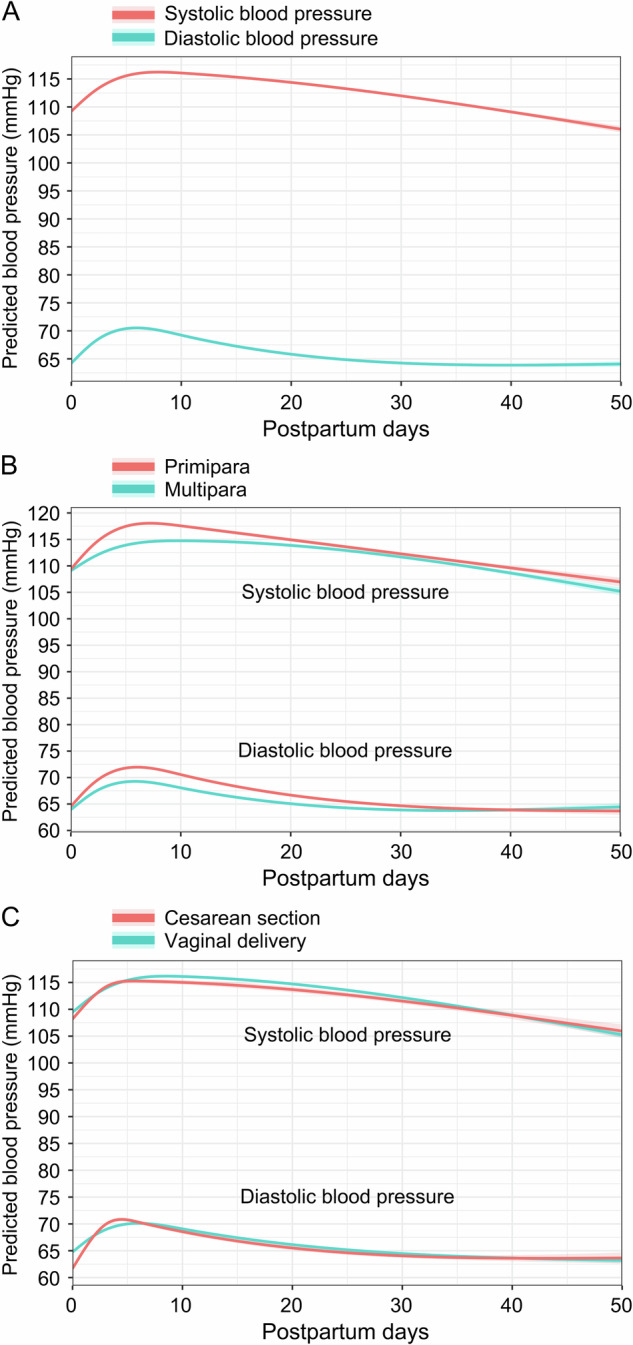
Model-estimated postpartum blood pressure trajectories. **A** Modeled postpartum blood pressure trajectories in low-risk, normotensive women. Red and blue lines indicate the model-estimated mean systolic and diastolic blood pressures, respectively, derived from linear mixed-effects models incorporating restricted cubic splines (df = 3). Shaded areas indicate 95% confidence intervals. **B** Postpartum blood pressure trajectories in nulliparous (red line) and multiparous (blue line) women. **C** Postpartum blood pressure trajectories stratified by the mode of delivery (red line, cesarean section; blue line, vaginal delivery)

Postpartum BP showed a clear temporal pattern (Fig. 4A). Both systolic and diastolic BP increased after delivery, peaked at approximately 5–7 days postpartum, and subsequently declined gradually over the following weeks. The parity-stratified postpartum BP trajectories are shown in Fig. 4B (nulliparous women are indicated with red lines and multiparous women with blue lines). Nulliparous women exhibited a greater increase in BP than multiparous women. Although the overall BP trajectories did not differ markedly between vaginal and cesarean deliveries, the timing of the peak BP varied slightly according to the delivery mode (Fig. 4C). BP returned to levels comparable to those observed in early pregnancy at approximately 4 weeks postpartum, and further converged to those observed in non-pregnant women by approximately 7 weeks postpartum.

## Discussion

In this large multicenter cohort study of normotensive women, we delineated BP trajectories spanning the estimated pre-pregnancy, pregnancy, labor, and postpartum phases. A notable and somewhat unexpected finding was that the model-estimated outpatient BP was higher in early pregnancy than in the estimated pre-pregnancy state, contrary to the conventional assumption that BP declines during early pregnancy. Thereafter, BP followed a well-established gestational pattern, increased markedly during labor, exhibited a transient peak in the early postpartum period, and ultimately returned to non-pregnant levels. By integrating longitudinal outpatient, intrapartum, and postpartum BP data, this study provides a comprehensive view of maternal BP dynamics across the peripartum continuum.

The observation that the estimated pre-pregnancy BP was lower than the outpatient BP measured in early pregnancy should be interpreted with caution because the pre-pregnancy BP was derived from matched non-pregnant women; nevertheless, this is a noteworthy finding. BP in early pregnancy has traditionally been thought to decrease physiologically from the pre-pregnancy baseline as a consequence of systemic vasodilation and reduced vascular resistance [1, 2, 17]. However, our findings suggest that, when assessed in routine outpatient settings, BP may transiently increase rather than decrease from the non-pregnant state to early pregnancy.

This finding is supported by external evidence. A U.S. cohort study that included 536 women without hypertensive disorders of pregnancy and incorporated direct pre-pregnancy measurements demonstrated an increase in systolic BP from the pre-pregnancy period to early pregnancy among nulliparous women [18]. Several mechanisms may explain the transient elevation in BP observed during early pregnancy. Early gestation is often accompanied by psychological stress and anxiety related to pregnancy recognition and the initiation of medical care, both of which may contribute to higher outpatient BP. Additionally, the clinical setting of early prenatal visits may accentuate the white-coat effect, reflecting increased sympathetic activity during medical evaluations. A previous study reported that early gestational BP increases occur predominantly among nulliparous women [18], suggesting that these increases may reflect heightened cardiovascular reactivity associated with psychological and emotional responses during the first pregnancy, rather than the physiological decline in BP typically expected in early gestation. However, an important consideration when interpreting these findings is the validity of the estimated pre-pregnancy BP derived from non-pregnant women. This estimate does not directly represent the true pre-pregnancy outpatient BP of each woman. Nevertheless, the postpartum BP gradually declined after delivery and converged toward the levels observed in non-pregnant women by approximately 2 months postpartum. This convergence suggests that the estimated pre-pregnancy BP provides a reasonably valid approximation of baseline BP at the population level.

The BP trajectories during pregnancy in this study closely mirrored the patterns established in previous studies, with gradual declines in both systolic and diastolic BP reaching a nadir at approximately 25 weeks of gestation, followed by an increase toward term [1, 2, 17]. Compared with home BP data from Japanese prospective cohorts [19, 20], the outpatient systolic BP in our study was consistently higher, whereas outpatient diastolic BP was comparable to or lower than home BP measurements, particularly during mid-pregnancy. Although the underlying reasons for these divergent trends remain unclear, differences in measurement environments, device characteristics, patient populations, and the timing of measurements may have contributed to this discrepancy.

One of the most clinically relevant findings is the magnitude of BP elevation observed during labor. Even among low-risk, normotensive women, systolic and diastolic BPs increased by approximately 15 and 13 mmHg, respectively, during the first stage of labor. These findings indicate that substantial BP elevation during labor is a component of normal physiology.

The mechanisms underlying this increase are likely multifactorial and involve sympathetic nervous system activation due to labor pain, increased catecholamine release, hemodynamic effects of uterine contractions, and maternal expulsive efforts [2, 7]. In clinical practice, intrapartum hypertension is associated with serious complications, including intracranial hemorrhage, hypertensive crisis, and eclampsia. In the present study, we found that, even among low-risk women, BP increased during labor to a greater extent than expected.

Postpartum BP exhibited a distinct temporal pattern, increasing after delivery, peaking around postpartum days 5–7 and gradually declining thereafter. Consistent with previous reports based on home BP measurements, BP decreased to levels comparable to those observed in early pregnancy by approximately 4 weeks postpartum [21] and returned to the levels observed in non-pregnant women by approximately 7 weeks postpartum. Clinically, these findings underscore the importance of BP monitoring during the first week postpartum, even in women who remain normotensive throughout their pregnancy. The early postpartum BP peak likely reflects multiple physiological mechanisms, including transient intravascular volume expansion due to the mobilization of interstitial fluid, sleep deprivation, psychological stress, breastfeeding-related neuroendocrine changes, and the use of medications such as uterotonics and nonsteroidal anti-inflammatory drugs [2, 9, 10]. The subsequent decline and convergence toward baseline further support the physiological nature of this pattern. However, as shown in Supplementary Fig. 1, the distribution of BP measurements during pregnancy and the postpartum period was not uniform. As this retrospective study was based on routinely collected clinical data, the timing of postpartum BP measurements varied across individuals and was subject to some degree of clustering and sparsity at specific postpartum time points. Therefore, the estimated postpartum BP trajectory may have been influenced by the nonuniform distribution of measurements and should be interpreted with caution. Nevertheless, the overall postpartum pattern was consistent with that reported in previous home BP studies, thereby supporting the reliability of the observed trajectory.

This study has several strengths. First, the large sample size enabled a detailed characterization of BP trajectories across the peripartum period in a large multicenter routine care population. Second, although previous prospective studies, including those based on home BP measurements, have provided important insights into BP trajectories during pregnancy and the postpartum period, these trajectories may be influenced by the characteristics of the population from which they were derived, including maternal age, body mass index, and other clinical backgrounds. In the present study, the application of linear mixed-effects models with restricted cubic splines allowed for flexible modeling of nonlinear BP patterns while accounting for interindividual variability, irregular measurement intervals, and maternal characteristics. This approach enabled the estimation of covariate-adjusted BP trajectories under specified conditions, rather than simply describing crude population averages. Third, the comprehensive temporal coverage from the estimated pre-pregnancy BP through pregnancy, labor, and postpartum recovery represents a major advancement over previous studies that focused on isolated periods. In particular, the inclusion of intrapartum BP data and the integration of BP measurements across the entire peripartum continuum are important novel contributions of this study.

However, this study has some limitations that should be considered when interpreting our findings. First, pre-pregnancy BP was approximated using an externally matched cohort of non-pregnant women rather than direct measurements obtained from the pregnant participants themselves. Although this approach provides a pragmatic method for generating a reference estimate, these values do not represent the true individual pre-pregnancy outpatient BP of pregnant women and should instead be interpreted as a contextual reference for understanding BP trajectories throughout pregnancy. Consequently, we could not definitively determine whether the true pre-pregnancy BP was lower than the BP measured during early pregnancy. Prospective studies including direct BP measurements before conception and during early pregnancy are required to clarify this relationship. Second, BP measurements were obtained under different conditions throughout the study period, including antenatal outpatient visits, hospital-based measurements during labor and the early postpartum period, post-discharge assessments, and health checkups in the non-pregnant reference population. Differences in measurement settings, device type, clinical context, body position, and timing may have introduced measurement-related variability. Therefore, some features of the observed BP trajectory should be interpreted with caution. Third, medication effects, including oxytocin administration during labor, postpartum use of nonsteroidal anti-inflammatory drugs and uterotonics, and epidural anesthesia, could not be fully accounted for. Finally, although the dataset was large, the study was retrospective in nature, and certain time points had a substantial proportion of missing data. Therefore, the potential impact of missingness on the results could not be fully evaluated.

In conclusion, this large cohort study provides a comprehensive description of BP trajectories across pregnancy, labor, and the postpartum periods. In analyses estimating pre-pregnancy BP using a matched cohort of non-pregnant women, our findings suggest the possibility of a transient elevation in BP during early pregnancy compared with the pre-pregnancy state. However, confirmation through prospective studies using direct preconception measurements is required. Defining the physiological BP patterns across the peripartum continuum may improve the clinical interpretation of intrapartum and postpartum BP changes.

## Supplementary information

Supplementary information

Supplementary Figure 1

## Data Availability

Data supporting the findings of this study are available from the corresponding author (TU) upon reasonable request and with permission from the Kishokai Medical Corporation and Central Clinic Group.
