# Supplementary material for: Blood pressure trajectories during pregnancy, labor, and postpartum period: a multicenter study in Japan
Source: Hypertens Res. 2026 Jun 10;49(8):2417–26. doi: 10.1038/s41440-026-02708-3 (PMC13437242; doi:10.1038/s41440-026-02708-3)
Supplement: Supplementary file 1 [file 41440_2026_2708_MOESM2_ESM.docx]

Supplementary Figure 1. Distribution of BP measurements during pregnancy and the postpartum period

(A) Number of BP measurements by gestational week during pregnancy. (B) Number of BP measurements by postpartum day. BP, blood pressure.
